# Supplementary material for: A novel curation system to facilitate data integration across regional citizen science survey programs
Source: PeerJ. 2020 Jul 29;8:e9219. doi: 10.7717/peerj.9219 (PMC7395600; doi:10.7717/peerj.9219)
Supplement: Supplemental Information 1 [file peerj-08-9219-s001.pdf]

North American Butterfly Association (NABA) Checklist 2nd edition (version 2.3) and updates (NABA 2016). This list is a standard for many popular field guides (Brock & Kaufman, 2006; Glassberg, 2017; Layberry, Hall & Lafontaine, 1998). This checklist contains 731 species and gives both scientific and common names. The first edition, based on Opler's (1992) taxonomic treatment in the Peterson *Field Guide to Eastern Butterflies* and Scott's (1986) *Butterflies of North America* was developed by NABA in 1992 as the first consensus checklist of North American butterfly names. The second edition is a revision by an appointed names committee which conservatively assessed each name from the first edition within the context of whether "conclusive evidence," based solely on published literature, indicated that the taxon should be treated differently. The NABA names committee provided a discussion of each potential name change (Cassie et al., 2001). Three interim reports have subsequently provided taxonomic updates (Caterino, Glassberg & Heraty, 2003; NABA names committee, 2015; NABA names committee, 2016).

Opler & Warren, 2005. These authors put together this species list (hereafter referred to as O&W) to showcase the nomenclature as known to that date for North American butterflies, and highlight nomenclatural problems. The list is built on previous standards: Ferris (1989) and Miller & Brown (1981), with conservative use of generic names largely following Opler (1992), Opler (1998), Opler (1999) and Opler, Stanford & Pavulaan (2000). Both authors agree upon almost all included species as based on both biological and phylogenetic species concepts (see Wheeler & Meier (2000) for discussion of species concepts). Higher taxonomic ranks (through family level) reflect the arrangements of Vane-Wright, de Jong & Ackery (1996) and Ackery et al. (1999). The list does not include subspecies names, although explanatory text for some taxa includes taxonomic affinities of sub-species populations. Opler & Warren's (2005) list comprises 784 species names. The list also includes an appendix of 74 species previously included as part of the North American butterfly fauna either erroneously or because of unsubstantiated or dubious documentation.

Pelham (2008). A complete catalog of scientific names for North American butterflies in the United States and Canada, including all synonyms and subspecies (we used the 2014 version, and referred to it in our text as Pelham). The 2014 list includes 820 species names, and 1571 subspecies names. Pelham's catalog is the most recently developed published taxonomic resource to date. Pelham continues to release revised versions on the Butterflies of America webpage (https://www.butterfliesofamerica.com/US-Can-Cat.htm), with the most recent revision published there April 22, 2019.
