# Supplementary material for: A novel curation system to facilitate data integration across regional citizen science survey programs
Source: PeerJ. 2020 Jul 29;8:e9219. doi: 10.7717/peerj.9219 (PMC7395600; doi:10.7717/peerj.9219)
Supplement: Supplemental Information 2 [file peerj-08-9219-s002.pdf]

**Flag type 1. Complex taxonomy; donor interpretation differs from receiver's.** Pairwise comparisons in the upper left quadrant of the *C. ladon* complex matrix in Fig. 3 occur between programs that use the same or compatible taxonomic interpretation of the NABA base list. The outcomes for pairwise comparisons in this quadrant are bi-directionally equivalent, that is, the outcome is the same when Receiver and Donor roles are reversed. For example, Michigan's compatible outcome for Iowa's request of *C. ladon* is *C. ladon/neglecta*; Iowa's compatible outcome for Michigan's request of *C. ladon/neglecta* is *C. ladon*. For these comparisons, we assume the data-user will interpret the taxonomic identities from the Receiver's perspective, however because there are so many alternative taxonomic interpretations and program exceptions to base taxonomy our tool returns flags for these comparisons to warn the user of other existing taxonomic interpretations that affect compatibility. At this point, our flags are very specific but future tool incarnations will have more generic flagging, with its purpose simply to alert the user of an issue.

Example:
Receiver list: Iowa; Taxon of interest: Celastrina ladon
Donor list: Ohio.
Tool output: Celastrina ladon, Celastrina neglecta
Flag: Proceed with caution. Since Iowa includes neglecta as a subspecies of C. ladon, data are compatible as long as both ladon and neglecta from Ohio are included together. Conflicting species concepts of C. ladon exist, which disagree on whether neglecta is a subspecies of C. ladon or whether these are distinct species. User should decide on appropriateness of combining these data.

**Flag type 2. Reverse requests not bidirectionally equivalent.** The upper right and lower left quadrants of the matrix in Fig. 3 show outputs for comparisons between programs based on different authorities. In the upper right quadrant the Receivers use NABA (or modified NABA interpretation), whereas the Donor lists use O&W or Pelham (these base lists interpret the *C. ladon* complex similarly). The reverse is true in the lower left quadrant where Receivers use O&W or Pelham and Donor's lists are based on NABA. The blue shaded cells in these quadrants (Fig. 3) indicate comparisons that do not have equivalent bi-directional outcomes. Often this is because one of the two programs' taxonomic perspective does not recognize the taxon at the species level, resulting in a zero match in one direction. The nature of the parent/child relationship in our tool makes the mapping file necessarily bi-directionally identical. Thus, in these cases, the tool's output reports the same non-zero outcome for both directions of the comparison, and a flag alerts the user to the conflict, explaining the perspective of all the different taxonomic interpretations. This alert allows the user to choose the appropriate outcome from the alternatives. An important element lacking in the tool, and a subject for future work, is our ability to consider the user's taxonomic perspective, which may be different from the donor or the receiver programs.

The difficulty of asymmetrical interpretations being treated as bidirectionally identical relationships must be weighed against the scalability afforded by the parent/child mapping structure. Specifically, this bidirectional symmetry allows for the ease with which our system allows a new monitoring program to be added to the mapping file. For our algorithm to work, all that is needed from each program is their checklist along with identification of any deviations from the authority they specify as their base. Thus as new regional monitoring programs are established, we can easily include their checklist into our tool, curating with each name their intended taxonomic usage by simply adding the mappings from the names on their project list to the equivalents on their base list.

Example:
Receiver list = 'Cascades.' Taxon = 'Celastrina lucia'
Donor list = 'MPG.'
Tool outcome: Celastrina echo
Flag: Proceed with caution. Conflicting taxonomic concepts of C. ladon complicate data combination in this case. User must assess compatibility. If user considers C. echo and C. lucia separate species from C. ladon, then there is no match in this list. If user considers echo and lucia subpsecies of ladon, data are compatible but only if both C. echo and C. lucia from Cascades is combined with C. echo from MPG.
